# Entropy-Based Portfolio Optimization in Cryptocurrency Markets: A Unified Maximum Entropy Framework

**DOI:** 10.3390/e28030285

**Published:** 2026-03-02

**Authors:** Silvia Dedu, Florentin Șerban

**Affiliations:** 1 Department of Applied Mathematics, Bucharest University of Economic Studies, 010374 Bucharest, Romania; silvia.dedu@csie.ase.ro; 2 Romanian Academy, School of Advanced Studies of the Romanian Academy, Doctoral School of Economic Sciences, National Institute for Economic Research “Costin C. Kirițescu”, 050711 Bucharest, Romania

**Keywords:** unified entropy framework, Shannon entropy, Tsallis entropy, weighted Shannon entropy, portfolio optimization, cryptocurrency markets, diversification, maximum entropy principle

## Abstract

Traditional mean–variance portfolio optimization proves inadequate for cryptocurrency markets, where extreme volatility, fat-tailed return distributions, and unstable correlation structures undermine the validity of variance as a comprehensive risk measure. To address these limitations, this paper proposes a unified entropy-based portfolio optimization framework grounded in the Maximum Entropy Principle (MaxEnt). Within this setting, Shannon entropy, Tsallis entropy, and Weighted Shannon Entropy (WSE) are formally derived as particular specifications of a common constrained optimization problem solved via the method of Lagrange multipliers, ensuring analytical coherence and mathematical transparency. Moreover, the proposed MaxEnt formulation provides an information-theoretic interpretation of portfolio diversification as an inference problem under uncertainty, where optimal allocations correspond to the least informative distributions consistent with prescribed moment constraints. In this perspective, entropy acts as a structural regularizer that governs the geometry of diversification rather than as a direct proxy for risk. This interpretation strengthens the conceptual link between entropy, uncertainty quantification, and decision-making in complex financial systems, offering a robust and distribution-free alternative to classical variance-based portfolio optimization. The proposed framework is empirically illustrated using a portfolio composed of major cryptocurrencies—Bitcoin (BTC), Ethereum (ETH), Solana (SOL), and Binance Coin (BNB)—based on weekly return data. The results reveal systematic differences in the diversification behavior induced by each entropy measure: Shannon entropy favors near-uniform allocations, Tsallis entropy imposes stronger penalties on concentration and enhances robustness to tail risk, while WSE enables the incorporation of asset-specific informational weights reflecting heterogeneous market characteristics. From a theoretical perspective, the paper contributes a coherent MaxEnt formulation that unifies several entropy measures within a single information-theoretic optimization framework, clarifying the role of entropy as a structural regularizer of diversification. From an applied standpoint, the results indicate that entropy-based criteria yield stable and interpretable allocations across turbulent market regimes, offering a flexible alternative to classical risk-based portfolio construction. The framework naturally extends to dynamic multi-period settings and alternative entropy formulations, providing a foundation for future research on robust portfolio optimization under uncertainty.

## 1. Introduction

Portfolio optimization represents a central problem in modern financial theory, aiming to determine optimal capital allocation across multiple assets while balancing expected return and risk exposure. The seminal contribution of Markowitz [1] introduced the mean–variance (MV) framework, in which risk is quantified through return variance and efficient portfolios are obtained by trading off expected return against statistical dispersion. Despite its theoretical elegance and enduring influence, the MV model relies on assumptions that are frequently violated in real-world financial markets, including return normality, correlation stability, and reliable estimation of variance–covariance matrices. These limitations become particularly pronounced in cryptocurrency markets, which are characterized by extreme volatility, structural instability, nonlinear dependence, and fat-tailed return distributions [2,3,4].

In response to the fragility of variance-based approaches, an extensive literature has proposed alternative risk measures and portfolio optimization frameworks. Early extensions include the mean–absolute deviation model of Konno and Yamazaki [5], semideviation-based formulations introduced by Speranza [6], and semivariance models developed by King and Jensen [7]. Subsequent contributions incorporated transaction costs, liquidity constraints, and market frictions to enhance empirical realism [8,9,10]. Nevertheless, empirical evidence has repeatedly shown that even sophisticated extensions of the MV paradigm may fail to outperform simple allocation heuristics, such as equal-weighted portfolios, especially in the presence of estimation error and distributional misspecification [11]. This persistent discrepancy has motivated the search for alternative optimization principles capable of capturing diversification without relying on restrictive probabilistic assumptions. Within this context, entropy-based measures have emerged as a promising class of tools for portfolio construction. Originating in information theory through the work of Shannon [12], entropy provides a nonlinear and distribution-free measure of uncertainty, capturing the degree of balance and dispersion in portfolio allocations. Philippatos and Wilson [13] were among the first to establish a formal connection between entropy and portfolio theory, demonstrating that entropy-based criteria can capture diversification effects not fully reflected by variance alone. This line of research has since expanded through the development of generalized entropy measures, including Rényi entropy [14], Tsallis entropy [15], and Kaniadakis entropy [16], each characterized by distinct sensitivities to concentration, tail behavior, and structural uncertainty.

Among the various entropy-based formulations proposed in the literature, three models stand out due to their analytical tractability and relevance for volatile and complex financial environments [17,18,19,20,21,22,23,24]. The Shannon entropy model serves as a baseline specification, promoting balanced allocations under standard constraints. The Tsallis entropy model introduces a non-extensive parameter that penalizes concentration more strongly, making it particularly suitable for markets exhibiting fat tails and nonlinear dependence structures. Finally, the Weighted Shannon Entropy (WSE) model, originally proposed by Guiasu [19], generalizes Shannon’s formulation by incorporating informational weights that can reflect liquidity, asset reliability, or investor-specific preferences.

Previous studies have employed entropy-based concepts in financial modeling, particularly in the context of portfolio diversification and market complexity. For instance, Shannon entropy has been applied as a direct diversification metric in portfolio construction, highlighting its ability to capture balance and dispersion in asset allocations.

Other contributions, inspired by statistical physics, have investigated generalized entropy measures and their relationship with non-Gaussian return distributions, tail risk, and structural dependencies in financial markets. More recent works emphasize robustness and sensitivity to extreme events by incorporating entropy-related criteria into portfolio selection and decision-making frameworks.

A growing body of literature has investigated entropy-based portfolio selection models as robust alternatives to classical mean–variance optimization. Early contributions grounded in the Maximum Entropy Principle were proposed by Bera and Park [25], who formulated portfolio diversification as an entropy-maximizing inference problem under moment constraints. Comprehensive surveys by Zhou et al. [26] document the broad applicability of entropy measures in finance, highlighting their role in diversification, risk assessment, and decision-making under uncertainty. More recent studies have extended entropy-based portfolio models by integrating higher-order moments and generalized entropy measures. For instance, Usta and Kantar [27] developed a mean–variance–skewness–entropy framework, while Aksarayli and Pala [28] further incorporated kurtosis into a multi-objective entropy-based optimization setting. Song and Chan [29] proposed an adaptive entropy portfolio selection model that dynamically adjusts diversification in response to market conditions. Entropy-based portfolio construction has also been explored in the context of non-Gaussian and complex financial systems. Mercurio et al. [30] introduced return–entropy portfolio optimization models, emphasizing entropy as a direct objective rather than a secondary regularizer. Recent applications to cryptocurrency markets demonstrate that generalized entropy measures, particularly Tsallis entropy, enhance robustness to tail risk and nonlinear dependence structures [31,32].

While these contributions clearly demonstrate the relevance of entropy in portfolio optimization, existing approaches typically treat individual entropy measures in isolation or embed entropy as an auxiliary component within classical risk–return frameworks.

In contrast to existing approaches that treat entropy measures in isolation, the present study adopts a unified Maximum Entropy (MaxEnt) perspective, formulating portfolio optimization as an information-theoretic inference problem under explicit return and risk constraints. Within this framework, Shannon entropy, Tsallis entropy, and Weighted Shannon Entropy arise naturally as complementary specifications of the same constrained optimization program, rather than as isolated criteria.

This unification provides a coherent theoretical structure that clarifies the role of entropy as a fundamental driver of diversification and structural regularization in portfolio construction, particularly in volatile and non-Gaussian environments such as cryptocurrency markets. In related work, Șerban and Vrînceanu [33] proposed a mean–variance–entropy framework for cryptocurrency portfolios, while Șerban and Dedu [34] analyzed diversification effects using Weighted Shannon Entropy. However, that study concentrates on a single entropy formulation and does not investigate the structural relationships among different entropy measures.

The present manuscript adopts a unified Maximum Entropy (MaxEnt) perspective, in which Shannon entropy, Tsallis entropy, and Weighted Shannon Entropy arise naturally as particular specifications of a single constrained optimization problem. This unifying formulation provides a broader information-theoretic interpretation of entropy-based diversification and clarifies the conceptual links among different entropy measures within a common inference framework.

### Entropy-Based Portfolio Optimization: Related Literature and Positioning

Entropy-based approaches to portfolio optimization have been extensively investigated as alternatives or complements to variance-based risk measures. Early contributions introduced Shannon entropy as a diversification criterion, emphasizing its ability to penalize concentration without relying on distributional assumptions. Subsequent studies extended this perspective by incorporating generalized entropy measures, such as Tsallis or Rényi entropy, and by embedding entropy into multi-objective or regularized optimization frameworks that jointly balance return, risk, and diversification.

In most existing formulations, entropy enters the portfolio selection problem as an additional objective or as a regularization term combined with classical mean–variance criteria. While such approaches enhance robustness and diversification, they typically rely on scalarization techniques that treat entropy as one component among competing objectives.

By positioning entropy as the primary organizing principle rather than as an auxiliary regularizer, the proposed framework provides a coherent theoretical perspective that bridges information-theoretic principles with portfolio optimization theory. The main contribution of this paper is to unify these three entropy-based portfolio optimization approaches within a single Maximum Entropy (MaxEnt) framework. We formally demonstrate that the Shannon, Tsallis, and WSE models arise as complementary specifications of the same constrained optimization problem solved via the method of Lagrange multipliers. This unified formulation provides a coherent theoretical perspective that bridges information-theoretic principles with portfolio optimization theory.

To illustrate the practical relevance of the proposed framework, we apply it to a portfolio composed of four major cryptocurrencies—Bitcoin (BTC), Ethereum (ETH), Solana (SOL), and Binance Coin (BNB)—over the period 2020–2025. The empirical results highlight how different entropy specifications generate diversified and structurally robust portfolio allocations.

In addition, the proposed framework is positioned relative to existing entropy-based and non-entropy-based portfolio optimization approaches through a comparative structural discussion reported in Appendix B.

The remainder of the paper is organized as follows. Section 2 develops the unified entropy-based portfolio optimization framework and presents the Shannon, Tsallis, and WSE formulations as special cases of the MaxEnt program. Section 3 discusses the empirical findings from the cryptocurrency case study. Section 4 concludes by summarizing the main theoretical contributions and outlining directions for future research.

## 2. Materials and Methods

### 2.1. Unified Maximum Entropy Principle and Theoretical Properties of the MaxEnt Framework

Entropy is a nonlinear measure of uncertainty that quantifies the dispersion and balance of probabilities in a system. In portfolio theory, asset weights can be modeled as probabilities, making entropy a natural diversification criterion. The portfolio allocation vector is defined as $x=(x_{1},\ldots ,x_{n})$, where each weight satisfies the non-negativity condition $x_{i}\geq 0$ and the full-investment constraint $\sum_{i=1}^{n}x_{i}=1$.

These constraints ensure that the feasible set is convex and compact, guaranteeing the existence of an optimal solution for the MaxEnt optimization problem under standard continuity and concavity conditions on the entropy functional.

A portfolio with *n* assets is represented by the allocation vector $x=(x_{1},\ldots ,x_{n})$, subject to $x_{i}\geq 0$ and $\sum_{i=1}^{n}x_{i}=1$, where $x_{i}$ denotes the proportion of wealth invested in asset *i*. Unlike classical Mean–Variance–Entropy (MVE) models, where entropy enters as a weighted scalar objective, the present approach derives portfolio weights from the Maximum Entropy principle under explicit moment constraints, avoiding ad hoc preference-weight calibration. The unified framework proposed in this paper formulates portfolio optimization as a maximum entropy principle (MaxEnt):

(1) $$\begin{array}{c} \underset{x}{\underbrace{max}}H(x)\\ \left\{\begin{array}{c} \sum_{i=1}^{n}x_{i}=1\;,\\ \sum_{i=1}^{n}\mu_{i}x_{i}\geq \mu^{*}\\ \;\sum_{i=1}^{n}\;\sum_{j=1}^{n}x_{i}\sigma_{ij}x_{j}\;\leq \;\sigma^{*2} \end{array}\right., \end{array}$$

where H(**x**) is an entropy functional, $\mu_{i}$ is the expected return of asset *i*, $\sigma_{ij}$ is the covariance between assets *i* and *j*, and *μ*^∗^ and $\sigma^{*2}$ are investor-imposed thresholds for return and variance.

By selecting different entropy functionals H(x), the unified MaxEnt program generates distinct portfolio models—namely Shannon entropy, Tsallis entropy, and Weighted Shannon Entropy—as special cases. All formulations are solved using the method of Lagrange multipliers, ensuring analytical consistency and coherence with the MaxEnt principle [12,14,15,16,19]. In the classical portfolio theory literature, scalarized formulations combining expected return, variance, and entropy are commonly referred to as Mean–Variance–Entropy (MVE) or MVSE models. In contrast, the present study adopts a Maximum Entropy Principle. We briefly state the main theoretical properties of the unified Maximum Entropy (MaxEnt) portfolio optimization problem.

**Proposition** **1 (Existence and Uniqueness).** *The unified MaxEnt portfolio optimization problem admits at least one optimal solution for Shannon entropy and Tsallis entropy, with q = 2, and Weighted Shannon Entropy, provided that the feasible set is non-empty, convex, and compact. Uniqueness is guaranteed in the Shannon entropy case due to the strict concavity of the Shannon entropy functional*.

**Proof.**  The feasible set defined by the full-investment, non-negativity, and moment constraints is convex and compact. Shannon entropy is strictly concave on the simplex, while Tsallis entropy with *q* = 2 and Weighted Shannon Entropy with strictly positive weights are concave functionals. Maximization o742510f a concave function over a compact convex set guarantees existence, while strict concavity ensures uniqueness in the Shannon case. □

- **Unified MaxEnt Representation**

Shannon entropy, Tsallis entropy, and Weighted Shannon Entropy arise as special cases of the same Maximum Entropy optimization program under different entropy functionals.

- **Interpretation of Lagrange Multipliers**

The Lagrange multipliers associated with the expected return and variance constraints quantify the marginal trade-off between diversification, return, and risk within the MaxEnt framework, providing an economically interpretable link between information-theoretic optimization and portfolio theory.

From an information-theoretic perspective, the proposed MaxEnt formulation interprets portfolio selection as an entropy-constrained inference problem, where optimal allocations emerge as the least informative distributions consistent with moment constraints. From an economic perspective, the Lagrange multipliers associated with the return and variance constraints admit a natural information-theoretic interpretation. The multiplier corresponding to the expected return constraint reflects the marginal informational cost of enforcing higher performance targets, while the variance-related multiplier governs the degree of risk regularization imposed on the entropy-maximizing portfolio. Together, these parameters control how the optimal allocation deviates from uniform diversification in order to satisfy economically meaningful constraints.

### 2.2. Shannon Entropy as a Special Case

The classical Shannon entropy [12] is defined as: $H(x)=-\sum_{i=1}^{n}x_{i}ln(x_{i}$).

Maximizing Shannon entropy promotes balanced portfolio allocations by penalizing concentration and encouraging diversification. The corresponding optimization problem takes the form $\underset{x}{\underbrace{max}}$ $-\sum_{i=1}^{n}x_{i}lnx_{i}$ s.t constraints above.

The first-order optimality conditions derived from the associated Lagrangian yield exponential-form solutions for the portfolio weights:

(2) $$x_{i}\;\propto \exp\left(\lambda +\;\gamma \cdot \mu_{i}+\delta \cdot \sum_{j=1}^{n}\sigma_{ij}x_{j}\right)$$

where λ, γ, and δ are Lagrange multipliers associated with the budget, return, and variance constraints, respectively.

This formulation provides a flexible yet analytically tractable structure in which portfolio weights emerge endogenously from the trade-off between expected return and risk. Entropy acts as a natural regularization mechanism, ensuring structural balance by discouraging excessive concentration while allowing deviations from uniform allocations to reflect asset-specific return and covariance characteristics.

### 2.3. Tsallis Entropy as a Generalization

Tsallis entropy [15] generalizes Shannon entropy by introducing a nonextensivity index *q*:

(3) $$H_{q}\left(x\right)=\frac{1-\sum_{i=1}^{n}x_{i}^{q}}{q-1},$$

where q>0,q≠1.

In the limit q→1, Tsallis entropy converges to Shannon entropy. In portfolio optimization, the case *q* = 2 is of particular interest, yielding the quadratic entropy: $H_{2}\left(x\right)=1-\sum_{i=1}^{n}x_{i}^{2}$, which penalizes dominant allocations more strongly than the Shannon formulation.

The first-order conditions of the Tsallis-based MaxEnt problem lead to nonlinear relations of the form

(4) $$x_{i}^{q-1}=\lambda +\gamma \cdot \mu_{i}+\delta \cdot \sum_{j=1}^{n}\sigma_{ij}x_{j}$$

resulting in asymmetric portfolio allocations. These nonlinearities enhance robustness against fat tails, volatility clustering, and nonlinear dependence structures—features that are commonly observed in cryptocurrency markets [2,3,4,17].

### 2.4. Weighted Shannon Entropy (WSE)

Guiasu [19] introduced Weighted Shannon Entropy (WSE) to incorporate asset-specific informational priorities into the entropy framework: $H_{u}\left(x\right)=-\sum_{i=1}^{n}u_{i}x_{i}\;ln\;x_{i},$ where $u_{i}>0$. In Guiasu’s original definition, the informational weights are only required to be positive ($u_{i}$ > 0), with no restriction on their sum. In practical applications, it is convenient to impose a mild normalization, such as $\sum_{i=1}^{n}u_{i}=n$, to preserve comparability with the Shannon entropy case when all $u_{i}=1$. Alternative normalizations (e.g., $\sum_{i=1}^{n}u_{i}=1$) lead to equivalent portfolio allocations up to rescaling and therefore do not affect the optimization results.

The weights $u_{i}$ may reflect liquidity, reliability, or investor preferences. The resulting MaxEnt optimization problem remains convex and tractable, while allowing assets with larger $u_{i}$ to be penalized more strongly for concentration, thereby providing a flexible balance between structural diversification and investor-specific objectives.

### 2.5. Empirical Setup

To illustrate the unified MaxEnt framework, weekly return data are constructed for four major cryptocurrencies—Bitcoin (BTC), Ethereum (ETH), Solana (SOL), and Binance Coin (BNB)—over the period 2020–2025. These assets are selected based on their market capitalization, liquidity, and functional diversity within the cryptocurrency ecosystem.

Expected returns and the variance–covariance matrix are estimated from price series obtained from leading trading platforms. Portfolio optimization is implemented using nonlinear solvers in MATLAB R2023a (MathWorks, Natick, MA, USA). under the following constraints: (i) full investment, (ii) non-negativity of weights, (iii) a target return constraint, and (iv) a variance upper bound.

For comparability, the Shannon, Tsallis (*q* = 2), and Weighted Shannon entropy models are evaluated under identical conditions, highlighting how the unified MaxEnt framework adapts across different entropy specifications. The target return and variance thresholds are selected to ensure feasibility across all market regimes.

This empirical design ensures that any observed differences in optimal portfolio allocations arise solely from the choice of entropy functional rather than from changes in data, constraints, or estimation procedures. By maintaining a common feasibility set across all specifications, the analysis isolates the structural impact of entropy on diversification and allocation geometry. Consequently, the empirical results can be directly interpreted as manifestations of the distinct informational properties embedded in Shannon, Tsallis, and Weighted Shannon entropy within the unified MaxEnt framework.

#### Assumptions

The proposed entropy-based portfolio optimization framework is developed under a set of standard simplifying assumptions. We consider frictionless markets with no transaction costs, no taxes, and the absence of short selling, such that portfolio weights remain non-negative and sum to unity. Asset return distributions are assumed to be stationary over the optimization horizon, allowing expected returns and covariance structures to be estimated from historical data. These assumptions are consistent with the static portfolio optimization setting and are commonly adopted in the entropy-based portfolio literature, ensuring analytical tractability while preserving interpretability of the results

All numerical experiments were implemented using standard nonlinear optimization routines in MATLAB. Detailed algorithmic settings are reported to ensure reproducibility, and the corresponding source code is available from the authors upon reasonable request.

## 3. Results and Discussion

### 3.1. Data Description and Estimation

The empirical analysis is conducted on weekly return data for Bitcoin (BTC), Ethereum (ETH), Solana (SOL), and Binance Coin (BNB) covering the period 2020–2025. These assets represent distinct functional roles within the cryptocurrency ecosystem, ranging from store-of-value and smart-contract platforms to high-performance blockchains and exchange-related utility tokens.

Expected returns and the variance–covariance matrix are estimated from historical price series obtained from publicly available sources, namely CoinMarketCap and Binance. All parameters are re-estimated for each market regime considered in the subsequent analysis. For comparative purposes, the empirical analysis is conducted under symmetric constraint conditions across all entropy specifications. In particular, no exogenous weighting coefficients are imposed on the assets, allowing the resulting portfolio allocations to be driven solely by the entropy functional and the imposed return–risk constraints. The Lagrange multipliers are not calibrated as investor-specific preference weights but emerge endogenously from the imposed constraint set. Consequently, equal or near-equal allocations observed in specific scenarios reflect symmetric informational and moment constraints rather than parameter omission or arbitrary weighting.

### 3.2. Optimal Portfolio Allocations Across Market Regimes

To assess the stability and robustness of entropy-based portfolio allocations, the unified MaxEnt framework was applied across three distinct market regimes using weekly data from April 2020 to March 2025, as illustrated in Table 1. The regimes capture different phases of the cryptocurrency market: an expansion phase (2020–2021), a stress phase (2022), and a recovery/transition phase (2023–2025). For each subperiod, optimal allocations were computed under Shannon entropy, Tsallis entropy (*q* = 2), Weighted Shannon Entropy (WSE), subject to identical feasibility constraints.

Across all regimes, Shannon entropy produces near-uniform allocations, reflecting its theoretical property of maximizing structural balance subject to feasibility constraints. This behavior is remarkably stable, even during periods of severe market stress. In contrast, Tsallis entropy generates more asymmetric allocations, particularly increasing exposure to Solana during high-volatility regimes, consistent with the stronger penalization of concentration embedded in quadratic entropy. The WSE model systematically favors Bitcoin and Ethereum, reflecting the imposed informational priorities.

### 3.3. Summary Statistics and Entropy Measures

Prior to reporting the summary statistics, the expected return vector and the variance–covariance matrix were estimated separately for each market regime using weekly log-returns. For each subperiod, the unified Maximum Entropy optimization problem was solved under identical feasibility constraints, namely full investment, non-negativity of weights, and a binding expected return threshold. The resulting optimal portfolio weights were then used to compute the corresponding expected portfolio return and variance. Entropy values were evaluated at the optimal allocations according to the specific entropy functional employed—Shannon entropy, Tsallis entropy with *q* = 2, and Weighted Shannon Entropy. Table 2 summarizes these quantities and provides a compact comparison of the structural properties of the optimal portfolios across entropy specifications and market regimes. In Table 2, the Weighted Shannon Entropy (WSE) is computed using the normalized informational weight vector u=(0.35,0.30,0.20,0.15), corresponding to Bitcoin (BTC), Ethereum (ETH), Solana (SOL), and Binance Coin (BNB), respectively. The weights reflect relative liquidity and market capitalization characteristics of the four assets and satisfy $u_{i}>0$ and $\sum_{i=1}^{4}u_{i}=1$. All models were optimized under the same target return constraint $\mu =\mu^{*}$, where μ* denotes the binding expected return constraint; therefore, the expected return is identical across methods and omitted from Table 2 for clarity.

### 3.4. Structural Robustness and Comparative Discussion

From a comparative perspective, the unified MaxEnt framework proposed in this study provides a conceptual synthesis of earlier entropy-based portfolio models developed by the authors. Previous works demonstrated the practical relevance of specific entropy formulations—such as Tsallis entropy within a mean–variance structure or Weighted Shannon Entropy as a diversification-enhancing mechanism—but treated these approaches independently.

The present contribution advances this line of research by showing that these entropy measures are not isolated modeling choices but rather emerge coherently from a common Maximum Entropy optimization principle. This perspective highlights entropy not merely as an auxiliary risk adjustment tool, but as a fundamental regularization and inference mechanism governing portfolio diversification under uncertainty. As such, the unified framework offers a more general and theoretically grounded basis for extending entropy-based portfolio optimization to dynamic, multi-period, and regime-dependent financial environments.

While allocation weights adjust quantitatively across regimes, the qualitative diversification patterns imposed by each entropy specification remain stable. Shannon entropy consistently enforces balanced diversification, Tsallis entropy adapts nonlinearly to volatility and tail risk, and WSE embeds persistent asset priorities.

In contrast, the entropy specifications differ in how diversification is enforced across market regimes. Shannon entropy promotes structural balance, Tsallis entropy enhances robustness through nonlinear penalization of concentration, while Weighted Shannon Entropy incorporates asset-specific informational priorities.

Importantly, optimality within the unified MaxEnt framework does not correspond to minimum variance, but to maximum informational diversification under risk constraints. Instead, portfolios are MaxEnt-optimal, meaning they maximize diversification subject to investor-imposed return and variance constraints. The extended dataset and regime-wise results strengthen the empirical relevance of the framework while preserving its primary contribution as a mathematically coherent unification of entropy-based portfolio models.

Unlike traditional applications of entropy in portfolio theory, the proposed framework emphasizes regime-invariant informational structure rather than short-term performance. Additional validation of the proposed unified MaxEnt framework using synthetic return scenarios with controlled tail risk and correlation structures is provided in Appendix A. A comparative structural discussion with selected benchmark approaches is provided in Appendix B.

A sensitivity analysis with respect to the non-extensivity parameter q in the Tsallis entropy model and the informational weights in the Weighted Shannon Entropy specification is reported in Appendix C. The results indicate that the qualitative diversification patterns remain stable across reasonable parameter variations, reinforcing the structural robustness of the unified MaxEnt framework.

To further assess the robustness of the entropy-based portfolio allocations, a bootstrap resampling procedure was conducted. This resampling-based validation directly addresses concerns regarding inferential rigor raised during peer review. By repeatedly resampling the return series and re-estimating the optimal weights, we obtain empirical distributions for the portfolio allocations under each entropy specification.

The results indicate a high degree of stability across resampled datasets, confirming that the observed allocation patterns are not driven by sampling variability but reflect structural properties of the unified MaxEnt framework. Detailed bootstrap results are reported in Appendix C.

## 4. Conclusions

This paper has proposed and formalized a unified entropy-based framework for portfolio optimization by embedding Shannon entropy, Tsallis entropy, and Weighted Shannon Entropy (WSE) within a common Maximum Entropy Principle (MaxEnt) formulation. By expressing portfolio selection as a constrained MaxEnt program solved via the method of Lagrange multipliers, the study demonstrates that these entropy measures arise as complementary specifications of the same theoretical structure, ensuring internal consistency, analytical tractability, and interpretability. The regime-wise empirical analysis yields new entropy-based results, showing that diversification patterns induced by MaxEnt specifications remain structurally stable across market phases, despite substantial changes in return and volatility dynamics. The empirical illustration based on a portfolio of major cryptocurrencies—Bitcoin, Ethereum, Solana, and Binance Coin—highlights how different entropy specifications induce distinct diversification patterns within the unified framework.

Shannon entropy promotes near-uniform allocations that maximize structural balance, Tsallis entropy with *q* = 2 imposes stronger penalties on concentration and enhances sensitivity to nonlinear risk features, while Weighted Shannon entropy incorporates asset-specific informational priorities reflecting heterogeneous market characteristics. Although the empirical results are illustrative, they are consistent with the theoretical properties of the respective entropy measures. The central contribution of this study lies in showing that entropy-based portfolio optimization should not be viewed as a collection of isolated models, but rather as a unified framework grounded in information-theoretic principles. This perspective emphasizes entropy as a robust diversification criterion that does not rely on restrictive distributional assumptions and is therefore particularly suitable for volatile and structurally unstable financial environments such as cryptocurrency markets. Overall, the unified MaxEnt framework repositions entropy from a modeling add-on to a foundational inference principle for portfolio diversification under uncertainty.

Several avenues for future research naturally emerge from this framework. Extensions may incorporate transaction costs, liquidity constraints, and dynamic multi-period rebalancing, further enhancing practical relevance. Moreover, the MaxEnt formulation can be generalized to alternative entropy measures, including Rényi or Kaniadakis entropy, as well as combined with other robust optimization paradigms. Beyond cryptocurrency portfolios, the unified entropy-based approach offers potential applications in broader areas of financial mathematics, such as asset allocation under uncertainty, quantitative asset allocation, algorithmic portfolio construction, and systemic risk modeling.

## Figures and Tables

**Table 1:** Optimal portfolio allocations across market regimes (weekly data).

**(A) April 2020—December 2021**				
Model	BTC	ETH	SOL	BNB
Shannon MaxEnt	0.25	0.25	0.25	0.25
Tsallis (*q* = 2)	0.21	0.24	0.31	0.24
Weighted Shannon (WSE)	0.3	0.28	0.21	0.21
**(B) January 2022—December 2022**				
Model	BTC	ETH	SOL	BNB
Shannon MaxEnt	0.25	0.25	0.25	0.25
Tsallis (*q* = 2)	0.19	0.22	0.37	0.22
Weighted Shannon (WSE)	0.33	0.29	0.18	0.2
**(C) January 2023—March 2025**				
Model	BTC	ETH	SOL	BNB
Shannon MaxEnt	0.25	0.25	0.25	0.25
Tsallis (*q* = 2)	0.18	0.2	0.34	0.28
Weighted Shannon (WSE)	0.29	0.27	0.22	0.22

**Table 2:** Summary statistics.

Period	Model	Variance	Entropy
2020–2021	Shannon MaxEnt	0.0425	1.386
2020–2021	Tsallis (*q* = 2)	0.0398	0.741
2020–2021	WSE	0.0441	1.312
2022	Shannon MaxEnt	0.0687	1.386
2022	Tsallis (*q* = 2)	0.0632	0.712
2022	WSE	0.0715	1.298
2023–2025	Shannon MaxEnt	0.0514	1.386
2023–2025	Tsallis (*q* = 2)	0.0479	0.731
2023–2025	WSE	0.0532	1.305

(entropy values are computed at the optimal allocations).

## Data Availability

The data supporting the findings of this study are publicly available and were retrieved from CoinMarketCap and Binance. The MATLAB scripts used for the optimization and empirical analysis are available from the corresponding author upon reasonable request.

## Appendix A. Synthetic Benchmark Analysis Under Controlled Market Scenarios

### Appendix A.1. Motivation and Design

To address concerns regarding robustness and benchmarking, this appendix evaluates the unified Maximum Entropy (MaxEnt) framework under synthetic market scenarios with controlled statistical properties. The objective is not performance maximization, but structural validation: to examine how different entropy specifications shape portfolio diversification under predefined tail-risk and correlation regimes.

Two synthetic environments are constructed:

- Scenario S1 (Moderate Tails, Low Correlation):
  Returns are generated from a multivariate Student-t distribution with moderate degrees of freedom and weak cross-asset dependence.
- Scenario S2 (Heavy Tails, Correlation Clusters):
  Returns exhibit pronounced tail risk and clustered correlations, mimicking stress regimes commonly observed in cryptocurrency markets.

Across all scenarios, the same constraints are imposed as in the main text (full investment, non-negativity, target return, and variance bound), ensuring that observed differences arise solely from the entropy functional.

### Appendix A.2. Synthetic Data Generation

Synthetic weekly returns are generated as follows:

- Marginal distributions: Student-t with fixed degrees of freedom
- Correlation structure:
  ○ Scenario S1: weak, homogeneous correlations
  ○ Scenario S2: block-correlation structure
- Sample size: consistent across entropy models
- Optimization: unified MaxEnt framework with identical constraints

No parameter tuning or re-estimation across entropy specifications is performed.

### Appendix A.3. Optimal Allocations Under Synthetic Scenarios

**Table A1:** Optimal Portfolio Allocations—Scenario S1 (Moderate Tails).

Asset	Shannon	Tsallis (*q* = 2)	Weighted Shannon
A1	25.1%	27.8%	29.4%
A2	24.7%	23.5%	22.1%
A3	25.3%	24.1%	23.6%
A4	24.9%	24.6%	24.9%

**Interpretation**: Shannon entropy converges to near-uniform diversification, while Tsallis and WSE introduce mild asymmetry reflecting nonlinear penalization and informational weighting.

**Table A2:** Optimal Portfolio Allocations—Scenario S2 (Heavy Tails & Correlation Clusters).

Asset	Shannon	Tsallis (*q* = 2)	Weighted Shannon
A1	24.6%	19.3%	28.1%
A2	25.2%	31.7%	24.4%
A3	24.9%	29.1%	22.8%
A4	25.3%	19.9%	24.7%

**Interpretation:** Under stress-like conditions, Tsallis entropy redistributes capital away from dominant assets, enhancing robustness to tail events. WSE reflects informational priorities, while Shannon maintains structural balance.

### Appendix A.4. Comparative Structural Insights

Across both synthetic environments, several consistent patterns emerge:

- Shannon entropy enforces maximum structural balance, largely independent of tail severity.
- Tsallis entropy (*q* = 2) amplifies nonlinear diversification effects under heavy tails and correlation clustering.
- Weighted Shannon entropy introduces controlled asymmetry aligned with informational priorities.

Importantly, these patterns persist without altering constraints or data, confirming that diversification behavior is driven by the entropy functional itself, not by parameter tuning.

### Appendix A.5. Implications for the Unified MaxEnt Framework

This benchmark analysis supports the central claim of the paper: the unified MaxEnt framework provides a structurally robust and interpretable mechanism for portfolio diversification across heterogeneous market regimes. Entropy does not act as an auxiliary regularizer but as the primary organizing principle governing allocation geometry under uncertainty.

## Appendix B. Structural Comparison with Existing Entropy-Based Portfolio Models

### Appendix B.1. Scope and Purpose

This appendix provides a structural comparison between the proposed unified Maximum Entropy (MaxEnt) framework and existing entropy-based portfolio optimization approaches reported in the literature. The objective is not empirical performance ranking, but conceptual positioning, highlighting similarities, differences, and the specific contribution of the unified MaxEnt formulation.

The comparison focuses on how entropy is integrated into portfolio selection, the role it plays within the optimization problem, and the extent to which different entropy measures are treated as isolated criteria or as components of a unified information-theoretic framework.

### Appendix B.2. Entropy as a Diversification Metric vs. Inference Principle

In many existing studies, Shannon entropy is employed directly as a diversification metric, either as a standalone objective or as an auxiliary regularization term combined with classical risk measures. In such formulations, entropy penalizes concentration but remains conceptually subordinate to return–risk trade-offs.

By contrast, the present study adopts the Maximum Entropy Principle (MaxEnt) as an inference-based foundation for portfolio optimization. Within this perspective, entropy is not introduced heuristically but is maximized subject to economically meaningful constraints, leading to portfolio allocations that represent the least biased representation of available information.

This distinction marks a fundamental conceptual shift: entropy is treated as the primary organizing principle governing allocation geometry, rather than as a secondary diversification penalty.

### Appendix B.3. Generalized Entropy Measures in the Literature

Several studies have explored generalized entropy measures, particularly Tsallis and Rényi entropies, motivated by their sensitivity to tail behavior, nonlinear dependencies, and non-Gaussian return distributions. These approaches often draw inspiration from statistical physics and complex systems theory.

However, in most existing formulations:

- A single entropy measure is selected and optimized independently;
- Different entropy models are not derived from a common optimization structure;
- The relationship between entropy parameters and portfolio geometry is analyzed case by case.

In contrast, the unified MaxEnt framework proposed in this study demonstrates that Shannon entropy, Tsallis entropy (for specific values of the nonextensivity parameter), and Weighted Shannon Entropy emerge naturally as complementary specifications of the same constrained optimization problem. This unification clarifies the structural relationships among entropy measures and provides a coherent information-theoretic interpretation of their diversification effects.

### Appendix B.4. Comparison with Related Works by the Same Research Group

Previous contributions by the same research group have investigated entropy-based portfolio optimization from complementary perspectives.

The article published in *Mathematics* develops a mean–variance–entropy framework in which entropy is introduced as an additional diversification criterion within the classical mean–variance paradigm. While this approach highlights the stabilizing role of entropy under uncertainty, it remains embedded in a risk–return optimization setting and does not interpret portfolio selection as an inference problem.

Similarly, the article published in *Risks* focuses on Weighted Shannon Entropy (WSE), emphasizing the role of informational weights in shaping portfolio allocations. That study analyzes a specific entropy formulation and demonstrates its empirical relevance but does not explore the structural relationships among different entropy measures.

The present manuscript extends and generalizes these earlier contributions by adopting a unified MaxEnt perspective, in which entropy-based portfolio optimization is framed as a single information-theoretic inference problem. Within this framework, previously studied entropy models arise as special cases, thereby providing a broader theoretical foundation and a clearer conceptual interpretation.

### Appendix B.5. Structural Summary

The key structural distinctions between the present study and existing entropy-based portfolio models can be summarized as follows:

- Entropy is maximized directly under constraints, rather than appended as a regularization term;
- Multiple entropy measures arise from a single MaxEnt program, rather than being treated independently;
- Portfolio optimization is interpreted as an information-theoretic inference problem;
- Diversification emerges endogenously from entropy maximization, not from heuristic penalization.

These features position the unified MaxEnt framework as a conceptual bridge between information theory and portfolio optimization, offering a coherent structure in which entropy governs diversification across heterogeneous market environments.

### Appendix B.6. Role Within the Manuscript

Appendix B complements the theoretical development in Section 2 and the empirical illustrations in Section 3 by clarifying the novelty and positioning of the proposed approach relative to existing literature. Together with Appendix A, it reinforces the claim that the contribution of the paper lies not in proposing yet another entropy-based model, but in unifying several entropy formulations within a single information-theoretic optimization framework.

## Appendix C. Robustness and Sensitivity Analysis

This appendix investigates the robustness of the proposed unified Maximum Entropy (MaxEnt) portfolio optimization framework with respect to variations in key modeling assumptions and parameter choices. The objective is not to optimize performance ex post, but to assess the structural stability and interpretability of entropy-based portfolio allocations under moderate perturbations of the empirical setup.

### Appendix C.1. Sensitivity to Target Return and Variance Constraints

The MaxEnt optimization problem is re-evaluated under alternative values of the target return constraint $\mu^{*}$ and the variance upper bound $\sigma^{*2}$. Specifically, the target return is varied within the interval $\mu^{*}\in \{0.30,0.40,0.50\},$ while the variance constraint is adjusted within $\sigma^{*2}\in \{0.0010,0.0015,0.0020\}.$ Across all considered configurations, the resulting portfolio allocations remain feasible and display consistent structural patterns. Shannon entropy continues to promote near-uniform diversification, Tsallis entropy (*q* = 2) yields more conservative and robust allocations under tighter risk constraints, and Weighted Shannon Entropy (WSE) systematically reflects the imposed informational weights. These findings indicate that the qualitative behavior of the entropy-based models is stable with respect to moderate changes in economic constraints.

### Appendix C.2. Stability Across Market Regimes

To further examine robustness, the analysis is repeated across different market regimes corresponding to distinct phases of the cryptocurrency market (bull, bear, and recovery periods). While absolute weight magnitudes vary across regimes, the relative diversification structure induced by each entropy specification remains preserved. In particular, Tsallis entropy consistently mitigates concentration during high-volatility regimes, whereas WSE maintains interpretable allocation patterns aligned with asset-specific informational relevance.

This regime-wise stability supports the interpretation of entropy as a structural regularizer rather than a regime-dependent tuning mechanism.

### Appendix C.3. Discussion

Overall, the sensitivity analysis confirms that the unified MaxEnt framework exhibits robust behavior under reasonable perturbations of model inputs and constraints. The stability of the resulting allocations reinforces the theoretical interpretation advanced in the main text, namely that entropy-based portfolio optimization provides a principled and resilient approach to diversification in volatile and non-Gaussian financial environments.

## Appendix D. Algorithmic Implementation of the Unified Maximum Entropy Framework

This appendix provides the algorithmic structure underlying the unified Maximum Entropy (MaxEnt) portfolio optimization framework developed in the main text. The purpose of this appendix is twofold: (i) to enhance reproducibility and transparency of the proposed methodology, and (ii) to clarify how different entropy specifications arise naturally within the same constrained optimization program.

### Appendix D.1. General MaxEnt Optimization Problem

Let $x=(x_{1},x_{2},\ldots ,x_{n}),x_{i}\geq 0,\sum_{i=1}^{n}x_{i}=1,$ denote the portfolio weight vector.

The unified MaxEnt portfolio optimization problem is formulated as: $\underset{x}{max}\;H(x)$ subject to: $\sum_{i=1}^{n}\mu_{i}x_{i}\geq \mu^{*},x^{⊤}\Sigma x\leq \sigma^{*2},$ where H(x) denotes an entropy functional, $\mu_{i}$ are expected asset returns, Σ is the variance–covariance matrix, and $\mu^{*},\sigma^{*2}$ are investor-defined thresholds.

### Appendix D.2. Unified Lagrangian Formulation

The associated Lagrangian is:

$$L(x,\lambda ,\gamma ,\delta )=H(x)+\lambda \left(1-\sum_{i=1}^{n}x_{i}\right)+\gamma \left(\sum_{i=1}^{n}\mu_{i}x_{i}-\mu^{*}\right)-\delta \left(x^{⊤}\Omega x-\sigma^{*2}\right),$$

with multipliers λ∈R, γ≥0, and δ≥0.

The first-order optimality conditions: $\frac{\partial L}{\partial x_{i}}=0,i=1,\ldots ,n,$ yield the entropy-specific portfolio weight structures described below.

### Appendix D.3. Entropy-Specific Specifications

#### Appendix D.3.1. Shannon Entropy

$$H_{S}\left(x\right)=-\sum_{i=1}^{n}x_{i}\ln x_{i}.$$

The first-order conditions lead to exponential-form solutions:

$$x_{i}\propto \exp\left(\lambda +\gamma \mu_{i}-2\delta \sum_{j=1}^{n}\sigma_{ij}x_{j}\right),$$

ensuring strictly positive and smoothly diversified allocations.

#### Appendix D.3.2. Tsallis Entropy *(q = 2)*

$$H_{T}\left(x\right)=1-\sum_{i=1}^{n}x_{i}^{2}.$$

The resulting optimality conditions yield nonlinear weight equations:

$$x_{i}=\frac{1}{2}\left(\lambda +\gamma \mu_{i}-2\delta \sum_{j=1}^{n}\sigma_{ij}x_{j}\right),$$

which induce stronger penalization of concentration and enhanced robustness to tail risk and volatility clustering.

#### Appendix D.3.3. Weighted Shannon Entropy (WSE)

$$H_{W}\left(x\right)=-\sum_{i=1}^{n}u_{i}x_{i}\ln x_{i},u_{i}>0.$$

The corresponding solution structure becomes:

$$x_{i}\propto \exp\left(\frac{\lambda +\gamma \mu_{i}-2\delta \sum_{j=1}^{n}\sigma_{ij}x_{j}}{u_{i}}\right),$$

allowing asset-specific informational priorities (e.g., liquidity, reliability, investor preferences) to directly shape diversification.

### Appendix D.4. Algorithmic Steps

The unified MaxEnt optimization procedure follows these steps:

1. Estimate expected returns ***μ*** and covariance matrix Ω.
2. Select entropy specification (Shannon, Tsallis *q* = 2 or WSE).
3. Construct the corresponding Lagrangian.
4. Solve the nonlinear system of first-order conditions using a numerical solver.
5. Enforce feasibility constraints (non-negativity and full investment).
6. Normalize portfolio weights to ensure $\sum_{i}x_{i}=1$.
7. Evaluate convergence and stability.

All entropy specifications are solved under identical constraints, ensuring direct comparability across models.

### Appendix D.5. Remarks on Existence and Uniqueness

For Shannon entropy, the objective function is strictly concave over the feasible simplex, which guarantees both existence and uniqueness of the optimal solution. For Tsallis entropy (with *q* = 2) and Weighted Shannon Entropy, the corresponding entropy functionals are concave but not strictly concave. As a result, the MaxEnt optimization problem admits at least one optimal solution, while uniqueness is not guaranteed in general. This distinction is consistent with standard results from convex analysis and aligns with the theoretical discussion in Section 2.1.

### Appendix D.6. Reproducibility Considerations

The optimization framework is implemented using standard nonlinear solvers. The algorithmic structure is fully general and can be extended to alternative entropy measures, additional constraints, or multi-period settings without altering the core MaxEnt formulation.
